# Plasmonic Polarization Rotation in SERS Spectroscopy

**DOI:** 10.1021/acs.nanolett.2c04461

**Published:** 2023-04-03

**Authors:** Xiaofei Xiao, Raymond Gillibert, Antonino Foti, Pierre-Eugène Coulon, Christian Ulysse, Tadzio Levato, Stefan A. Maier, Vincenzo Giannini, Pietro Giuseppe Gucciardi, Giancarlo Rizza

**Affiliations:** † Technology Innovation Institute, P.O. Box 9639, Building B04C, Masdar City, Abu Dhabi, United Arab Emirates; ‡ CNR-IPCF, Istituto per i Processi Chimico-Fisici, Messina I-98158, Italy; § LSI, Institut Polytechnique de Paris, CEA/DRF/IRAMIS, CNRS, Ecole polytechnique, Route de Saclay, Palaiseau 91128, France; ∥ Centre for Nanoscience and Nanotechnology, CNRS, Université Paris-Saclay, Palaiseau 91140, France; ⊥ School of Physics and Astronomy, Monash University, Clayton, Victoria 3800, Australia; # The Blackett Laboratory, Imperial College London, London SW7 2AZ, United Kingdom; ∇ Chair in Hybrid Nanosystems, Nanoinstitute München, Faculty of Physics, Ludwig-Maximilians-Universität München, 80539 München, Germany; ○ Instituto de Estructura de la Materia (IEM-CSIC), Consejo Superior de Investigaciones Científicas, Serrano 121, 28006 Madrid, Spain; ● Centre of Excellence ENSEMBLE3 sp. z o.o., Wolczynska 133, Warsaw 01-919, Poland

**Keywords:** SERS, Raman scattering, SEROA, optical activity, plasmons, metasurfaces, metallic nanostructures

## Abstract

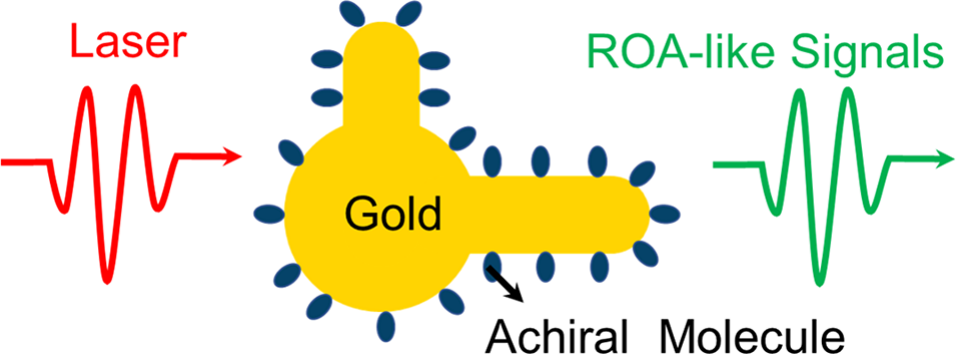

Surface-enhanced Raman optical activity (SEROA) has been extensively investigated due to its ability to directly probe stereochemistry and molecular structure. However, most works have focused on the Raman optical activity (ROA) effect arising from the chirality of the molecules on isotropic surfaces. Here, we propose a strategy for achieving a similar effect: i.e., a surface-enhanced Raman polarization rotation effect arising from the coupling of optically inactive molecules with the chiral plasmonic response of metasurfaces. This effect is due to the optically active response of metallic nanostructures and their interaction with molecules, which could extend the ROA potential to inactive molecules and be used to enhance the sensibility performances of surface-enhanced Raman spectroscopy. More importantly, this technique does not suffer from the heating issue present in traditional plasmonic-enhanced ROA techniques, as it does not rely on the chirality of the molecules.

Surface-enhanced Raman spectroscopy (SERS) uses plasmonic enhancement to amplify the Raman signal.^1−5^ The enhancements achieved through SERS are massive, making it one of the most sensitive techniques available.^1^ Enhancement of the polarization along small gaps^6^ and polarization rotation as a function of the excitation wavelengths have been observed.^7^ Obtaining similar enhancements in the polarization rotation of Raman scattering could be extremely beneficial.^8−10^

In previous works, metallic nanoparticles and the nanogaps in particle clusters have been utilized to enhance the weak ROA signal^11^ from chiral molecules absorbed on the surface of those particles or in the gaps,^12−14^ where electric field hot spots exist.^15,16^ The majority of researchers have focused on studying the polarization difference in the intensity of Raman scattering from chiral molecules on isotropic surfaces, with the common understanding being that the ROA signal arises from the intrinsic properties of the Raman tensor of the analyte (the chirality of molecules). However, it is also crucial to consider the orientation of the molecule with respect to the metallic nanoparticle surfaces and the chiral properties of the nanoparticles, as these can also influence the Raman signal. Despite this, the molecular orientation dependence of ROA signals is often overlooked. Furthermore, traditional plasmonic-enhanced ROA techniques can suffer from a commonly reported issue where heating in SERS substrates can reduce the chirality of molecules, leading to a decrease in the ROA effect.^17^

In this work, we introduce chiral Raman scattering engineering using the chirality of the plasmonic structures instead of the intrinsic chirality of the active molecules, allowing in this way to analyze Raman-inactive molecules using the well-known sensitivity of SERS. Specifically, we study how a linearly polarized pump generates rotated Raman scattering polarized in a different plane, similar to the well-known Faraday effect obtained using magnetic fields. This effect is investigated by exploiting the coupling of optically inactive molecules, such as methylene blue (MB), with anisotropic plasmonic metasurfaces. Our goal is to extend the ROA potential to inactive molecules as an additional factor in studying composition and molecular structures. To achieve this, we aim to design plasmonic systems capable of obtaining and controlling strong ROA-like signals from inactive molecules and fine-tuning the chiroptical phenomena to the frequencies of interest. This is similar to that observed from chiral molecules on an isotropic surface, but here the change in Raman light polarization arises from the anisotropy of the metallic nanostructures and their interaction with the molecule. Consequently, this technique does not suffer from the heating problem commonly associated with traditional plasmon-enhanced ROA techniques, which is related to the chirality of the molecules.^17^ This effect can extend the ROA potential to inactive molecules, providing an extra factor in studying composition and molecular structures.

We first designed anisotropic gold nanoparticles, which we refer to as a “nanoclock” due to their shape, and then investigated their optical activity properties. One such property is their ability to convert linearly polarized light into elliptically polarized light. Figure 1 shows a schematic of the nanoclock array. The Au nanoclock structure consists of a disk and two arms, which is convenient for tunability by changing arm lengths and the angle between them. As shown in Figure 1, the period, disk diameter, long arm length, short arm length, arm width, and thickness of the Au nanoclock structure are represented by Λ, *D*, *L*_1_, *L*_2_, *W*, and *H*, respectively.

**Figure 1:**
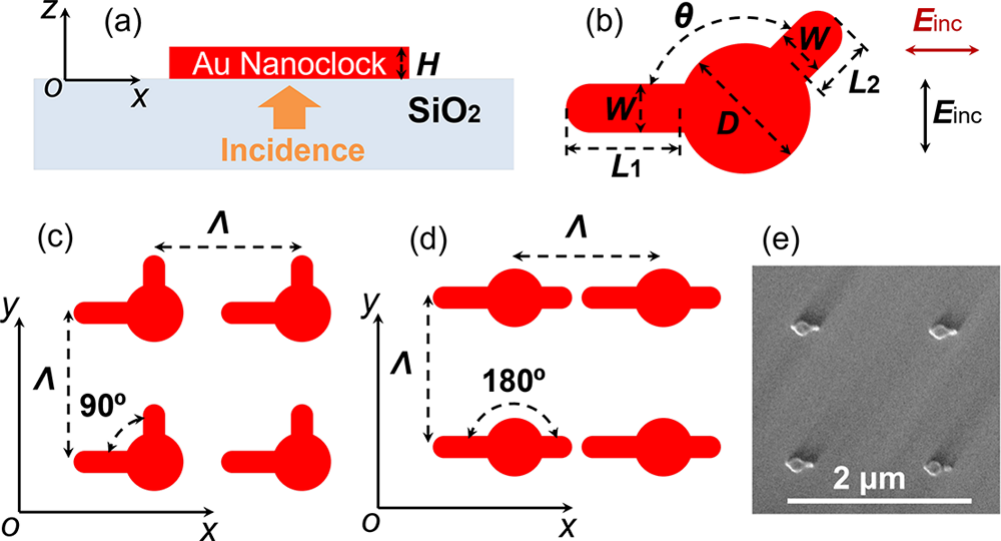
Schematic of the nanoclock array. (a) Au nanoclock structures are placed on the silica substrate (*n* = 1.45). *H* represents the thickness of the Au nanoclock structure. (b) Schematic of a single Au nanoclock particle. Each unit cell consists of a disc of diameter *D*, a long arm of length *L*_1_ and width *W*, and a short arm of length *L*_2_ and width *W*. The angles between the two arms are 90 and 180°, as shown in (c) and (d). The incoming beam is incident from the substrate side, normal to the surface. Light can impinge with polarization perpendicular (black arrow) and parallel (red arrow) to the long arm of the nanostructure, as shown in (b). The period in both *x* and *y* directions is denoted by Λ and has a value of either Λ = 500 nm or Λ = 1500 nm. The samples with the configuration in (c) are labeled as CL 90° 500 and CL 90° 1500, while those with the configuration in (d) are labeled as CL 180° 500 and CL 180° 1500, depending on the period. (e) Scanning electron microscopy image of the Au nanoclock array, when Λ = 1500 nm and θ = 180°.

To simplify our analysis, we have fixed some parameters as follows: *D* = 160 nm, *L*_1_ = 120 nm, *L*_2_ = 60 nm, *W* = 50 nm, and *H* = 50 nm. The ends of both arms are rounded, as shown in Figure 1b. An incident beam illuminates the structure from the substrate side, normal to the surface of the substrate. Two samples with Λ = 500 nm and Λ = 1500 nm are considered. The incident light can be polarized either perpendicular (vertical polarization) or parallel (horizontal polarization) to the long arm of the nanostructure. Two values (90 and 180°) of the angle (θ) between two arms are considered, as shown in Figure 1c,d. Scanning electron microscopy images of the fabricated Au nanoclocks are shown in Figure 1e and Figure S16, which confirms the rounded ends of both arms, consistent with the design parameters.

We begin by examining the linear optical response of the nanoclock arrays fabricated with a period of 500 nm (labeled as CL 90° 500 and CL 180° 500, respectively). In Figure 2, we present both the experimental and simulated extinction spectra. For experimental measurements, we used an XploRa plus Raman spectrometer (Horiba Jobin Yvon) in the transmission mode with normal incidence and linear polarization. The spectra were recorded with the incident electric field polarized perpendicular (black lines) and parallel (red lines) to the long arm of the nanostructure.

**Figure 2:**
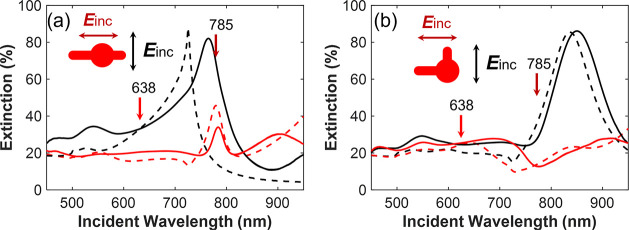
Experimental extinction spectra of the nanoclock arrays (a) CL 180° 500 and (b) CL 90° 500 recorded under two incident polarization states as labeled by arrows. Solid and dashed lines are experimental and simulated data, respectively. The vertical one-way arrows in the panels correspond to the incident excitation wavelengths. Insets: schematics of the structures.

We observe a distinct plasmonic resonance at a wavelength of 764 nm for the vertical polarization of the CL 180° 500 case (Figure 2a) and another resonance at a wavelength of 850 nm for the vertical polarization of the CL 90° 500 case (Figure 2b). Some resonances observed in the spectra, including dips and peaks, are plasmonic lattice modes resulting from the coupling of single plasmon resonances with Rayleigh anomalies due to the array period.^18−20^ However, horizontal polarization always exhibits less pronounced resonances due to the fundamental (dipole) resonances being at longer wavelengths than those in our measurements. In contrast, the fundamental mode resonance in the vertical polarization is visible in the wavelength range of interest.^18,19^ Additionally, the nanoclock length in the *y* direction is shorter for the CL 180° 500 case, resulting in a shorter wavelength resonance compared to the CL 90° 500 case, as expected.

To confirm our interpretation based on lattice modes, we investigated the single Au nanoclock particle in four configurations using finite-difference time-domain simulations. The results are shown in Figure 3, which shows the theoretical extinction cross section and the charge distribution at different resonance wavelengths on the top of the structures. It is observed that the shapes of the experimental extinction spectra of the array differ from the theoretical extinction cross sections of single nanoclocks. Additionally, the extinction cross section depends on both the structure and the incident polarization. The charge distribution indicates that the predominant resonance in each case corresponds to the dipole mode of the disk, the dipole mode of the arm, or the combination of the two modes. While the strongest extinction peaks are due to the dipole modes, other weaker resonances correspond to higher-order modes. Figure S1 shows the corresponding intensity enhancement (|**E**|^2^/|**E**_0_|^2^).

**Figure 3:**
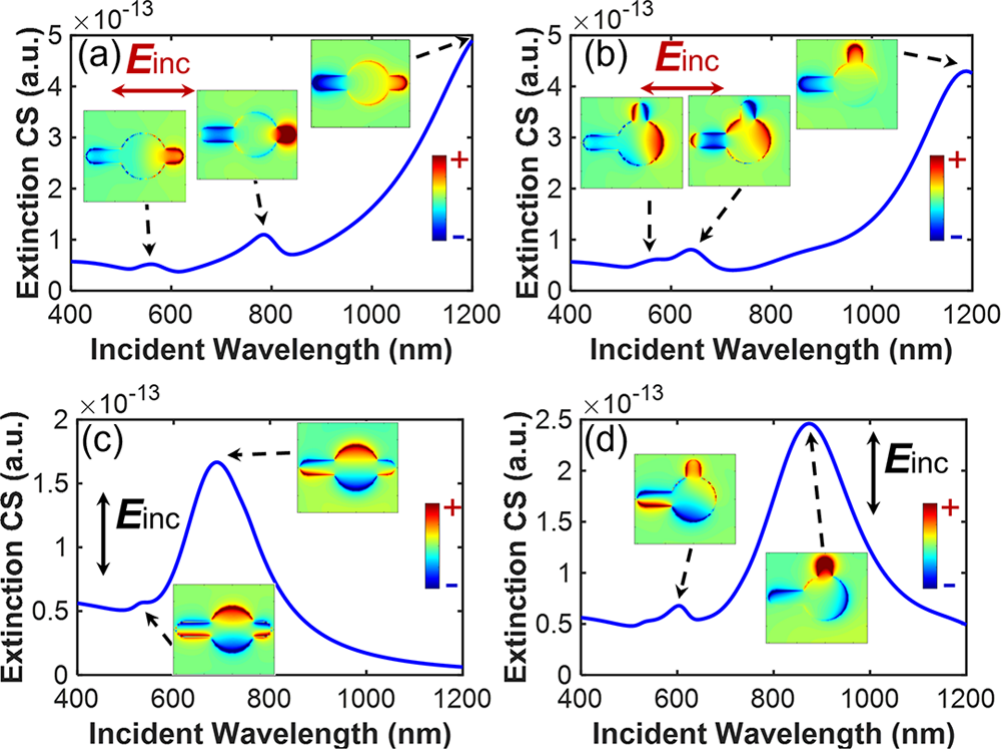
Theoretical results of the extinction cross section (CS) for a single Au nanoclock particle in four configurations. The corresponding distributions of *z* components of the electric field (*E*_*z*_) at the resonances, which correspond to charge distribution, are also provided. The fields are monitored in a plane located 1 nm above the structures and perpendicular to the incident wave vector. The corresponding wavelengths used in each plot are as follows: (a) 559, 784, and 1200 nm, (b) 564, 640, and 1188 nm, (c) 534 and 689 nm, and (d) 603 and 847 nm. The shape of the Au nanoclock particles is the same for θ = 180° in (a) and (c), while the shape is the same for θ = 90° in (b) and (d). The arrows indicate the polarization of the incidence. Horizontal polarization is used in (a) and (b), while vertical polarization is used in (c) and (d).

The location of hot spots that dominate the enhancement depends on the incident angle, polarization, and wavelength. Referring to Figure 3 and Figure S1, we can recognize the modes supported in each resonance peak. In Figure 3a, the mode at the wavelength of 1200 nm is a dipole resonance of the entire structure, resulting in the corresponding region with high enhancement covering the vicinity of the entire structure. At a wavelength of 784 nm in Figure 3a, the field corresponding to the higher-order mode is concentrated in the region around the long and short arms. In Figure 3c, the dominant resonance is the dipole mode of the disk at the wavelength of 689 nm. The mode of the combined region of the disk and the short arm is predominant in Figure 3d when the wavelength is at 847 nm. When the modes are fully excited, Raman scattering from the molecules in those regions is tremendously enhanced. Consequently, the polarization felt by the molecules located in the hot spots reflects the polarization of the local field. The Raman scattering in disordered systems is, in principle, randomly polarized, but only the scattering with the same polarization as the local field can efficiently couple with the nanostructures and be scattered away. This is the key to obtaining a ROA-like effect.

The next step involves a theoretical study of plasmonic excitation in the arrays. In Figure S4, we present the extinction spectra (1 – *T*, where *T* denotes the transmittance) for arrays of Au nanoclock particles (Λ = 500 nm). Numerical simulations were conducted using a finite-difference time-domain method for linearly polarized light at normal incidence. The dashed lines correspond to diffraction conditions (Rayleigh anomaly) in both air and substrate given by , where *i* and *j* are arbitrary integers and *n* = 1 (red lines for air) or *n* = 1.45 (green lines for substrate).^18,20−22^

The analytical predictions are consistent with the numerical results, as shown in Figure S4. Interestingly, the simulated spectra predict the presence of resonances near the Rayleigh anomaly (at a longer wavelength) when (*i*,*j*) = (0,1) or (1,0) and *n* = 1.45. This phenomenon arises from the collective mode of the localized plasmon resonances linked to individual nanoparticles.^18,22^ Compared to plasmonic resonances in isolated nanoparticles (as shown in Figure 3 and Figure S4), such lattice modes possess a considerably narrower bandwidth and a sharper response. The theoretical extinction spectra agree well with the experimental spectra, as illustrated in Figure 2. The slight discrepancies could be due to the potential nanofabrication defects and/or imperfections in the permittivities of the gold and substrate used for the simulation.

Let us focus on the ability of these structures to induce elliptically polarized light from linearly polarized light, as observed in optical activity experiments. In Figure 4, we present the polarization analysis for nanoclock arrays with a period of Λ = 500 nm and θ = 90°, which was performed by studying the far-field component of the transmitted light (see Figure 1). Elliptical polarization is induced when linearly polarized light is incident on the array at normal incidence. To characterize the polarization, an angle of polarization α and a polarization ratio can be defined from the electric field components, as shown in the inset of Figure 4a. Figure 4 reveals that the induced elliptical polarization properties depend on various factors such as the wavelength, the dimensions of the nanoclocks, and the polarization of the incident field. Left- or right-elliptical polarization can be generated, with the chiral plasmonic response of the metasurface introducing orthogonally polarized components. These components are responsible for the strong ROA-like signals from inactive molecules, as explained in the next section.

**Figure 4:**
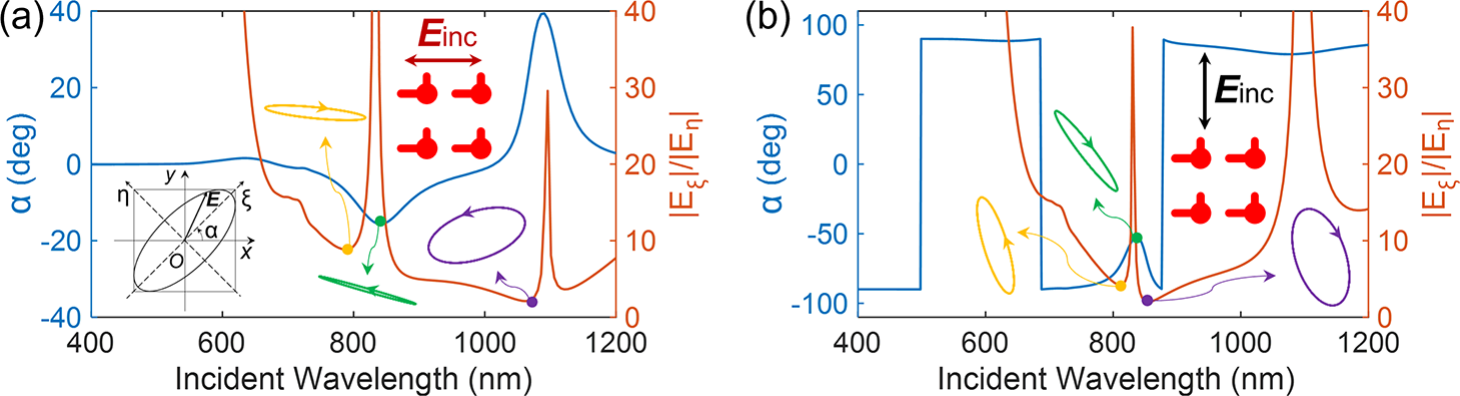
Far-field polarization calculation (transmission mode) for nanoclock particle arrays (θ = 90°) with a period of Λ = 500 nm. The polarization of the normal incidence is represented by arrows in (a) and (b). The light beam is impinging from the substrates (see Figure 1). The inset in (a) is a schematic of the elliptical polarization. α denotes the ellipse major axis angle (blue curve, left axes), and *E*_ξ_/*E*_η_ denotes the major/minor axis ratio (brown curve, right axes). The elliptical polarization is shown for representative wavelengths, including 780, 840, and 1062 nm in (a) and 811, 837, and 855 nm in (b).

We turn our attention to the Raman spectra of MB dye adsorbed onto the anisotropic plasmonic structures to investigate the Raman polarization rotation induced by these structures. The characteristic Raman shift of MB dye is in the range of 350–1700 cm^–1^. We evaluate the SERS enhancement factors for the structures on the arrays (CL 180° 500 and CL 90° 500) as well as for individual particles (CL 180° 1500 and CL 90° 1500) by using unpolarized scattering light at excitation wavelengths of 638 and 785 nm, respectively. The enhancement factors, which are given in Table S1, are obtained by dividing the raw SERS intensity measured in our samples by the Raman intensity of MB deposited on a flat glass substrate for the 1622 cm^–1^ band. This approach provides a rough quantification, since the data are not normalized according to the relative surface area of gold nanostructures, leading to a significant undervaluation of the actual enhancement factors.

Then we perform polarization-sensitive experiments by using polarized incidence and collecting polarized Raman signals. The co-polarization (the excitation and collection have the same polarization) and cross-polarization (the excitation polarization is perpendicular to the collection polarization) SERS signals are compared for different configurations. In general terms, for linearly polarized excitation, the Raman depolarization ratio of a molecular sample is determined as^23^
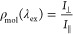
1where *I*_⊥_ and *I*_∥_ are the measured intensity of the Raman signal polarized orthogonal and parallel to the excitation polarization, respectively, and λ_ex_ denotes the excitation wavelength.

Without nanoantennas, we expect to approximately have a depolarization ratio of 0.5 at an excitation wavelength of 638 nm.^6^ However, considering the plasmonic and reradiation effects, we observed that the SERS depolarization ratio could be larger than 1 for both excitation wavelengths in our experiments. This confirms that the plasmonic structure induces strong Raman polarization rotation.

Assuming that the SERS signal mainly comes from bright resonances (and not dark resonances), we can use the extinction spectra to predict SERS enhancements. This is a rough approximation, but it simplifies the analysis. To compare the measured and predicted SERS depolarization ratios, we use the extinction (Ext = 1 – *T*) to calculate the SERS depolarization ratio at a given wavelength λ_R_ via the relation^1^
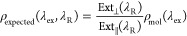
2where Ext_⊥_ and Ext_∥_ denote the extinction signal with the polarization orthogonal and parallel to the excitation polarization at the given wavelength, respectively. In this formula, we relate the far field with the Raman-scattering enhancement, which is a crude approximation because the Raman scattering is linked to the enhancements of the near fields.^24,25^ However, this gives us a good starting point because it allows us to compare experimental and numerical results.

We can predict the SERS depolarization ratio based on the measured and simulated extinction, which we refer to as sample prediction and simulation prediction. The experimental SERS depolarization ratio can be obtained by calculating the intensity ratio of the SERS signal (*I*_⊥_/*I*_∥_). We plot the predictions and the experimental SERS depolarization ratio as a function of the Raman scattering wavelength for the SERS bands at 437, 765, 1388, and 1620 cm^–1^. Results for both excitation wavelengths are shown in Figure 5 and Figure S5. The agreement between the sample prediction and the simulation prediction is good, which is expected from the good agreement between the experimental and simulated extinction spectra in Figure 2. For the case in Figure 5a, the disagreement can be explained by the shift between the experimental and simulated extinction spectra. The experimental SERS depolarization ratio at the SERS bands agrees reasonably well with the predictions with the excitation wavelength of 638 nm.

**Figure 5:**
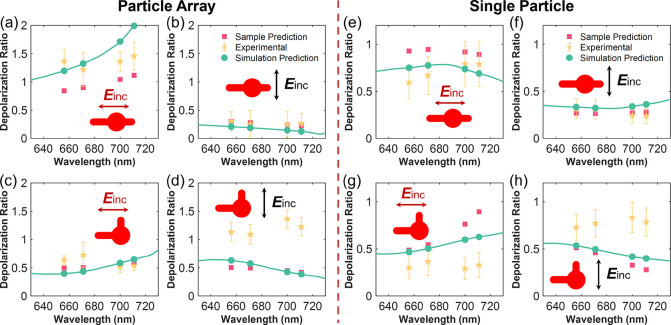
SERS depolarization ratio for nanoclock particle arrays with (a–d) period Λ = 500 nm (particle array) and (e–h) period Λ = 1500 nm (single particle). Orange stars denote the experimental depolarization ratio, while the sample prediction (red squares) and the simulation prediction (green dots and curves) are also plotted. The excitation wavelength is 638 nm. The insets shows the structures and the incident polarizations for each case.

It is important to note that the SERS depolarization ratio can vary significantly for the same molecule depending on various factors such as polarization, excitation wavelength, and configuration. This underscores the significant impact that plasmonic nanostructures can have on Raman signals. The SERS depolarization ratios shown in Figure 5 demonstrate that chiral Raman scattering can be induced by the anisotropy of the plasmonic structure instead of the intrinsic optical activity of active molecules, allowing for the analysis of Raman-inactive molecules using the well-known sensitivity of SERS. Moreover, this technique does not suffer from the heating issues associated with traditional plasmonic-enhanced ROA techniques^17^ because the ROA-like response observed in this study is not due to the chirality of the molecules. Consequently, the same phenomenon will occur even if the structure of the molecule is altered at high temperatures. This SEROA-like effect enables us to design plasmonic structures that yield strong ROA-like signals from achiral molecules and tailor the chiroptical phenomena to desired frequencies, making it an attractive strategy for the SEROA technique.

The results for individual nanoclocks on CL 180° 1500 and CL 90° 1500 samples are presented in Figure S6. It can be observed that the single structure yields results similar to those for the array, although the SERS signal becomes noisier for individual nanoclocks, as shown in Figures S5 and S6.

In summary, this work describes a strategy for achieving enhanced Raman scattering polarization rotation using plasmonic metasurfaces with chiral nanostructures. By combining SERS measurements and electromagnetic field calculations, we show that asymmetric plasmonic structures (nanoclocks) can interact with light in a complex manner, leading to high ROA-like effects. The polarization dependence of the SERS signal is in good agreement with the predictions obtained from the simulated and measured extinctions. These results indicate that, although the optical properties of the molecules are intrinsically inactive, chiral Raman scattering can be induced by engineering the anisotropy of the plasmonic structures, thereby enabling analysis of Raman-inactive molecules using the well-known sensitivity of SERS. Furthermore, this technique does not suffer from the heating problem commonly associated with traditional plasmon-enhanced ROA techniques that are related to the chirality of the molecules. Such a technique could extend the potential of ROA techniques to inactive molecules and enhance the sensitivity performance of the SERS.

## Supporting Information Available

Experimental section, details of experimental setup and Raman measurement, theoretical results of the extinction cross section of a single Au nanoclock particle, local intensity enhancement of the near field, raw SERS enhancement factors, theoretical results of extinction spectra for Au nanoclock particle arrays, SERS depolarization ratios for different samples and excitation wavelengths, comparison between theoretical extinction spectra and absorption spectra for Au nanoclock particle arrays, far-field polarization ellipse calculation for nanoclock particle arrays, scanning electron microscopy images of Au nanoclock array (PDF)
