# Supplementary material for: Plasmonic Polarization Rotation in SERS Spectroscopy
Source: Nano Lett. 2023 Apr 3;23(7):2530–5. doi: 10.1021/acs.nanolett.2c04461 (PMC10103303; doi:10.1021/acs.nanolett.2c04461)
Supplement: Supplementary file 1 — nl2c04461_si_001.pdf [file nl2c04461_si_001.pdf]

Supporting Information: Plasmonic Polarization Rotation in SERS Spectroscopy

Xiaofei Xiao,[*,†,∇] Raymond Gillibert,[‡,∇] Antonino Foti,[‡] Pierre-Eugène Coulon,[¶] Christian Ulysse,[§] Tadzio Levato,[†] Stefan A. Maier,[‖,⊥,#] Vincenzo Giannini,[†,@,△] Pietro Giuseppe Gucciardi,[‡] and Giancarlo Rizza[¶]

†*Technology Innovation Institute, P.O. Box 9639, Building B04C, Masdar City, Abu Dhabi, United Arab Emirates*

‡*CNR-IPCF, Istituto per i Processi Chimico-Fisici, Messina I-98158, Italy*

¶*LSI, Institut Polytechnique de Paris, CEA/DRF/IRAMIS, CNRS, Ecole polytechnique, Route de Saclay, Palaiseau 91140, France*

§*Centre for Nanoscience and Nanotechnology, CNRS, Université Paris-Saclay, Palaiseau 91140, France*

‖*School of Physics and Astronomy, Monash University, Clayton Victoria 3800, Australia*

⊥*The Blackett Laboratory, Imperial College London, London SW7 2AZ, United Kingdom*

#*Chair in Hybrid Nanosystems, Nanoinstitute München, Faculty of Physics, Ludwig-Maximilians-Universität München, 80539 München, Germany*

@*Instituto de Estructura de la Materia (IEM-CSIC), Consejo Superior de Investigaciones Científicas, Serrano 121, 28006 Madrid, Spain*

△*Centre of Excellence ENSEMBLE3 sp. z o.o., Wolczynska 133, Warsaw 01-919, Poland*

∇*Contributed equally to this work*

E-mail: Xiaofei.Xiao@tii.ae

Experimental Section

Materials and Methods

Sample Fabrication and Surface Functionalization of a Gold Nanoclock Array. Arrays of gold nanoclocks were fabricated on silica glass using electron beam lithography. First, the fused silica substrate was spin-coated with a 140 nm thick positive-tone polymethyl methacrylate (PMMA) resist (950K A4) layer and baked at 180 °C for 5 min. Then, the nanostructures were defined with an electron beam exposure, followed by the PMMA development in a standard 1:3 methyl isobutyl ketone/isopropanol (MIBK/IPA) solution (for 40 s). A 50 mn thick gold layer was deposited at 5 Å/s by thermal evaporation, and the lift-off process was performed in acetone as a final step. Although the adhesion layer can significantly improve the adhesion of the gold film to the glass, it is detrimental to plasmonic enhancements. In our fabrication, we did not use any adhesion layer. Instead, careful treatment was required to avoid the shedding of gold nanostructures. The cross-sectional images of the nanostructures were collected using a high-resolution scanning electron microscope (Magellan 400L, Thermofisher). The observation parameters were 1kV for 0.6pA, and the snapshot shooting mode was used. Functionalization was done by dipping the sample for 2 h in a 10^{-4} M concentrated Methylene Blue (MB) solution prepared in distilled water.

Optical Measurements

SERS measurements were performed using an XploRa plus Raman spectrometer (Horiba Jobin Yvon) with a 100× objective with a numerical aperture of 0.9, using the 1200 lines/mm grating for 638 nm and 600 lines/mm grating for 785 nm (see Figure S9). In both cases, a 300 µm pinhole was used. The slits at the entrance of the spectrometer were set to 100 µm. The laser power was reduced by using a neutral density filter. The excitation polarization could be turned 90° using a half-wave plate. It should be noted that many instrument components, such as the grating, can affect the interpretation of the polarization response

in a Raman measurement.[S1] We eliminate these potential issues by checking the incident polarization and calibrating the polarization response of the spectrometer in our measurements (details can be found below), guaranteeing the correct interpretation of the polarization response. When measuring nanoparticle arrays with a period of 500 nm, the laser spot covered multiple particles, whereas, for the 1500 nm period, the laser was focused on an individual nanoparticle. Extinction measurements were performed using the same spectrometer equipped with the 600 lines/mm grating (without the edge filter) in the transmission configuration, illuminating the sample from below with polarised light at normal incidence through the glass substrate and collecting the extinction signal using a 5× objective with 0.1 NA. For each configuration, five spectra at different positions were measured. The depolarization ratio of MB on gold film is 0.5 at an excitation wavelength of 638 nm and 0.33 at an excitation wavelength of 785 nm.[S2]

Numerical Simulations

Numerical simulations are carried out using three-dimensional finite-difference-time-domain (FDTD, Lumerical) simulations. The simulated structure is an Au nanoclock consisting of a disc and two arms. The silica substrate has a refractive index of 1.45, and the gold permittivity data are obtained from Johnson and Christy model.[S3] In the extinction cross-section simulation for a single Au nanoclock particle, perfectly matched layers (PMLs) are applied in all directions of the simulation space. An analysis group located inside the total-field scattered-field source calculates the extinction cross-section. For the simulation of the extinction spectra for Au nanoclock particle arrays, one unit cell is simulated using periodic boundary conditions on the left, right, front, and back of the simulation region, while the PMLs were applied at the top and bottom of the simulation region. To simulate the near-fields, two monitors were used. One monitor is used to obtain the z-component of the electric field (which corresponds to the charge distribution) in a plane 1 nm above the structures and perpendicular to the incident wave vector. The other monitor is used to monitor the electric

intensity through the center of the Au structure and perpendicular to the incident wave vector. The incidence wavelength is in the range of 400 nm and 1200 nm. For calculating the far-field polarization ellipse, the polarization ellipse analysis group in Lumerical FDTD is used.

Details of Experimental Setup and Raman Measurement

SERS polarization measurements are carried out using the polarization options of our XPLORA spectrometer, whose schematic of the optical paths and components is illustrated in Figure S9. The linear polarization of the lasers (638 nm and 785 nm) can be rotated by a halfwave plate. In the sample plane, we can therefore switch between two orthogonal polarization states (called pol V and pol H, see Figure S9). We have verified by means of a polarization analyzer and a photodiode that at both wavelengths, the light coming out of the microscope objectives is linearly polarized with a ratio $> 100 : 1$ for both polarization states H and V. The back-scattered SERS light is collected with the same objective used for light focusing and sent to the detection section consisting of an interchangeable set of diffraction gratings and a CCD camera. Polarization analysis is carried out by means of polarization analyzers with mutually orthogonal axes oriented parallel to the excitation polarization directions H and V. We can therefore excite our sample with two orthogonal linear polarization states (H or V) and, for each of them, detect the intensity of the parallel- (HH or VV) and the cross-polarized (HV or VH) component of the Raman/SERS field. Indeed, both the mirrors driving the light to the detector (generally oriented at 45° with respect to the beam direction) and the diffraction gratings used for spectral analysis have a polarization-dependent optical response. The polarization response of the Raman filter is less relevant given the quasi-orthogonal incidence/transmission geometry adopted in our setup. The polarization response of these elements consists of a dichroic behavior, i.e., they reflect with different

efficiency for the light polarized in the H and V states. The dichroism of the gratings, in particular, depends on the number of lines per mm, blazing angle, and central wavelength at which it is used. If not taken into account, these effects can induce severe artifacts in the interpretation of the measurements.

In order to get rid of these instrumental effects, a polarization scrambler or a further halfwave plate can be put along the optical detection path.[S1] In our case, this operation is much complicated by the closed architecture of the spectrometer, preventing any free access to the optical paths. Therefore, we have calibrated the polarization response of our spectrometer by using an unpolarized light source as a sample (Figure S10). The white light coming from the LED of an iPhone is acquired in the same experimental configuration used for SERS measurements (including objective, Raman filter, and grating), switching the polarization analyzer from polarization state H to polarization state V. This allows us to evidence any instrumental-induced dichroism causing the two H- and V-spectra to differ in intensity, as a function of the wavelength. Finally, we can define a calibration function to compensate for these instrumental effects.

Calibration measurements were carried out in both experimental configurations used for SERS at 785 nm with a 600 lines/mm (l/mm) grating and 638 nm with a 1200 l/mm grating. These laser/grating combinations were found to give the smallest instrumental artifacts, as shown in the following.

785nm excitation

Figure S11 (top plot) shows the spectra of the LED source when the analyzer is set in the two polarization states (H, green line; V, orange line). They show the different polarization responses of the spectrometer equipped with a 600 l/mm grating in the Raman shift range between 50 and 1750 cm^{-1} with respect to 785 nm (788 - 910 nm). This is the range used for SERS measurements. The instrument is more sensitive when the analyzer is set in the V polarization state with respect to the H polarization state. Differences range from 18% (at

800 cm^{-1}) to 30% (at 1750 cm^{-1}), as evidenced in the plot of the V/H signal ratio (Figure S11 , bottom). Although not dramatic, this sensitivity difference between the two polarization states produces artifacts that can be well observed, but also compensated, when measuring the depolarization ratio of a standard reference sample such as CCl_4. CCl_4 is a reference for polarized Raman polarization,[S1] featuring a symmetric vibration mode polarized parallel to the excitation field polarization and two depolarized asymmetric vibration modes exhibiting strong scattering in both the parallel- and cross-polarized states. For liquid samples in which molecules are randomly oriented, the depolarization ratio of the symmetric mode is expected to be $\rho_{\mathrm{sym}} = 0$, while the depolarization ratio of both the asymmetric modes is $\rho_{\mathrm{asym}} = 0.75$.

In Figure S12, we plot the parallel- (pol V, blue line) and cross-polarized (pol H, green line) Raman spectra of CCl_4 acquired at 785 nm with excitation polarization along the V direction. In both spectra, we observe strong scattering from the two F_2 asymmetric vibrations at 216 and 312 cm^{-1}, whereas the symmetric A_1 vibration signal at 452 cm^{-1} is strongly suppressed in the cross-polarized spectrum (pol H, green line). We calculate the depolarization ratios of the different modes by subtracting the background, fitting the peaks with Lorentzian profiles, and taking the ratio between the maximum peak intensities derived from the fitting parameters. Using the spectra obtained, notably, the depolarization ratio of the asymmetric modes turns out to be $\rho_{\mathrm{asym}} \sim 0.57$, i.e., 24% lower than what was expected. This is indeed the consequence of the higher sensitivity of the instrument towards the parallel-polarized (pol V) signal with respect to the cross-polarized one (pol H), highlighted above, that depletes the depolarization ratio. We can compensate for this effect by rescaling (i.e., dividing) the parallel-polarized (pol V) spectrum with respect to the V/H polarization response curve of the instrument (Figure S11, bottom plot). We, therefore, obtain a "corrected spectrum" (Figure S12, orange line) that is used to recalculate the depolarization ratios, which now are:

$$\rho_{\mathrm{asym}} = 0.76 \pm 0.02 \tag{1}$$

for both the asymmetric modes at 216 and 312 cm^{-1}, and

$$\rho_{\text{sym}} = 0.0077 \pm 0.0001 \quad (2)$$

for the symmetric mode at 452 cm^{-1}. Here the depolarization ratio is notably of the order of 10^{-3}. Both values are now in agreement with the predictions. In order to take into account these effects, the SERS spectra of MB have been all rescaled using the same calibration curve.

638nm excitation

The same measurements were carried out for the experimental configuration used for laser excitation at 638 nm, combined with a 1200 l/mm grating. The spectra of the unpolarized LED source in the polarization states V (orange line) and H (green line) are shown in Figure S13 (top). The H/V intensity ratio curve is plotted in Figure S13 (bottom). It is seen that the dichroic response of the instrument is less marked at 638 nm than at 785 nm and quasi-negligible. The H/V ratio oscillates around 1 by ±10% in the full spectral range. In the range of the Raman modes of MB, restricted to 420 - 1640 cm^{-1}, we expect an even smaller instrumental influence on the depolarization factors, of the order of 5%.

Again, to ensure minimal polarization-related artifacts occur with excitation at 638 nm and to compensate for them, we acquired Raman spectra of CCl_4. The laser is polarized in the V-direction. Figure S14 shows the parallel- (VV, blue line) and the cross-polarized (VH, orange line) Raman spectra. Subsequently, we rescaled the cross-polarized (VH) spectrum with respect to the instrumental H/V polarization response curve. Figure S15 shows the minimal differences between the as-acquired VH spectrum (blue line) and the corrected one (orange line).

Based on the rescaled spectrum, we calculate the depolarization ratios at 638 nm as

follows:

$$\rho_{\text{asym}} = \begin{cases} 0.74 \pm 0.01 & @216\text{cm}^{-1} \\ 0.75 \pm 0.01 & @312\text{cm}^{-1} \end{cases}$$

for the two asymmetric modes, and

$$\rho_{\text{sym}} = 0.0050 \pm 0.0003 \tag{3}$$

for the symmetric mode at 452 cm^{-1}. Again here the depolarization ratio is notably of the order of 10^{-3}. Both values are in agreement with the predictions.

In order to take into account these effects, even if minimal, the SERS spectra of MB have been all rescaled using the calibration curve, prior to calculating the depolarization ratios.

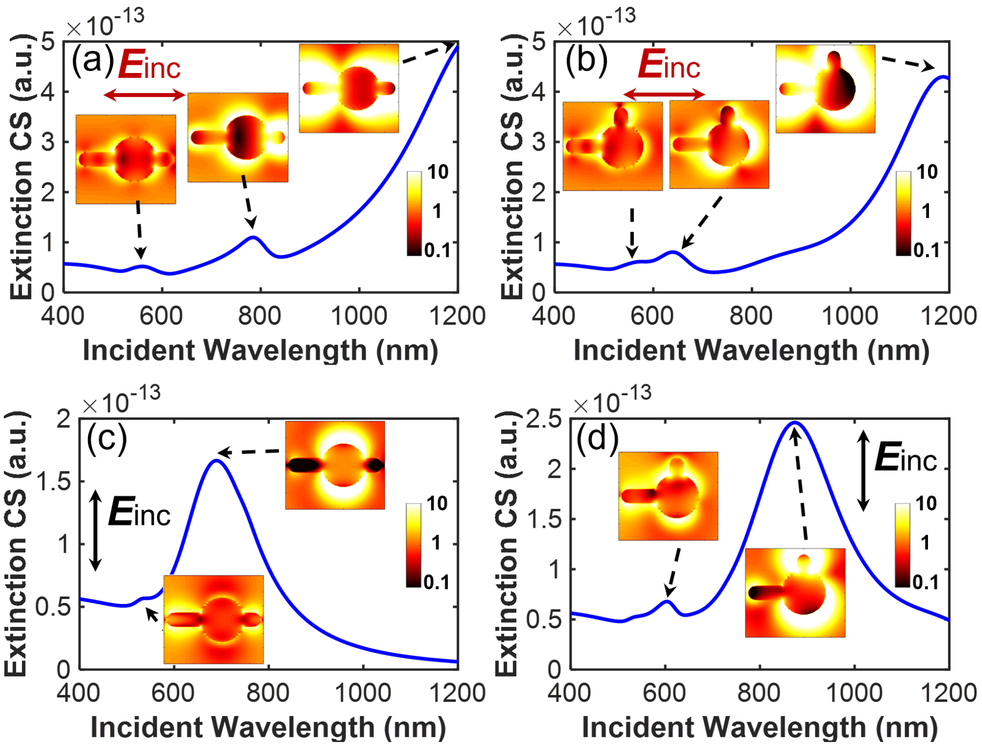

Figure S1: Theoretical results of the extinction cross-section (CS) of a single Au nanoclock particle with four configurations. The local intensity enhancement ($|\mathbf{E}|^2/|\mathbf{E}_0|^2$) (in logarithmic scale) corresponding to resonances is also provided. The monitors used for the plots are in a plane passing through the center of the Au structure and perpendicular to the incident wave vector. The wavelength used in each plot is as follows: (a) 559 nm, 784 nm, and 1200 nm, (b) 564 nm, 640 nm, and 1188 nm, (c) 534 nm and 689 nm, and (d) 603 nm and 847 nm. The near-field plots show that the shape of the Au nanoclock particles is the same ($\theta = 180°$) for (a) and (c), while the shape ($\theta = 90°$) in (b) and (d) is the same. The arrows represent the polarization of the incidence. Horizontal polarization is used in (a) and (b), while vertical polarization is used in (c) and (d).

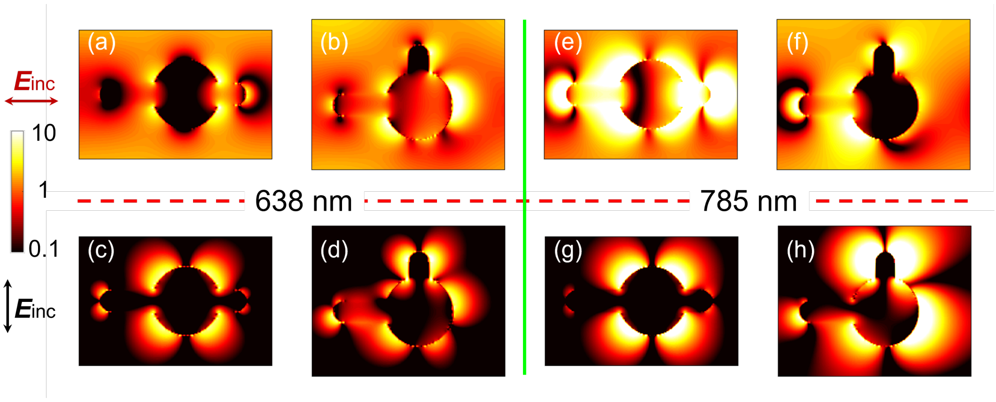

Figure S2: Local intensity enhancement of the x-component of the near-field ($|E_x|^2/|E_0|^2$) (in logarithmic scale) at two different excitation wavelengths: $\lambda_0 = 638\,\text{nm}$ ((a)-(d)) and $\lambda_0 = 785\,\text{nm}$ ((e)-(h)). The monitors used to obtain the data are located in a plane through the center of the Au structure and perpendicular to the incident wave vector. The polarization of the incidence is represented by the arrows. Horizontal polarization is used in (a), (b), (e), and (f), while vertical polarization is used in (c), (d), (g), and (h). The period $\Lambda_0 = 500\,\text{nm}$ is considered for all configurations.

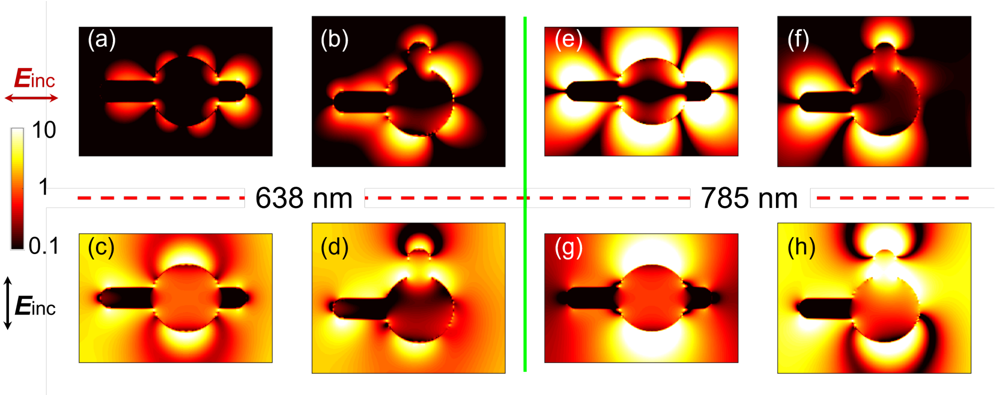

Figure S3: Local intensity enhancement of the y-component of the near-field ($|E_y|^2/|E_0|^2$) (in logarithmic scale) at two different excitation wavelengths: $\lambda_0 = 638\,\text{nm}$ ((a)-(d)) and $\lambda_0 = 785\,\text{nm}$ ((e)-(h)). The monitors used to obtain the data are located in a plane through the center of the Au structure and perpendicular to the incident wave vector. The polarization of the incidence is represented by the arrows. Horizontal polarization is used in (a), (b), (e), and (f), while vertical polarization is used in (c), (d), (g), and (h). The period $\Lambda_0 = 500\,\text{nm}$ is considered for all configurations.

Table S1: Raw SERS enhancement factors for all configurations with an unpolarized collection. The incident polarization is defined as horizontal (vertical) polarization when the incident polarization is parallel (perpendicular) to the long arm.

	Structure	Excitation wavelength (nm)	Excitation polarization	Enhancement factor (error ∼50%)
180°, Grating	A500	785	V	1.5×10^2
	A500	785	H	0.75×10^2
90°, Grating	B500	785	V	3.5×10^2
	B500	785	H	1×10^2
180°, Single	A1500	785	V	1.5×10^1
	A1500	785	H	1.5×10^1
90°, Single	B1500	785	V	4×10^1
	B1500	785	H	1×10^1
180°, Grating	A500	638	V	5×10^1
	A500	638	H	4×10^1
90°, Grating	B500	638	V	4×10^1
	B500	638	H	3×10^1
180°, single	A1500	638	V	5.5×10^0
	A1500	638	H	2×10^0
90°, Single	B1500	638	V	3.5×10^0
	B1500	638	H	3.5×10^0

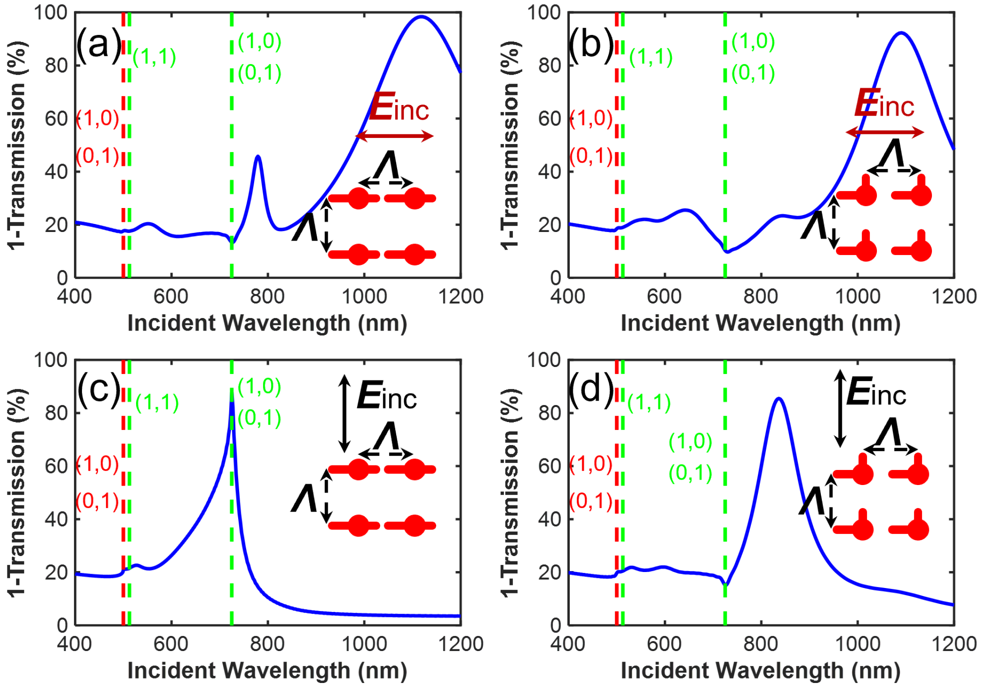

Figure S4: Theoretical results of extinction spectra $(1-T)$ for Au nanoclock particle arrays. The period $\Lambda = 500\,\mathrm{nm}$ is the same for all four configurations, also depicted in the insets. The shape of the Au nanoclock particles is the same for (a) and (c), while the shape in (b) and (d) is the same. The arrows represent the incidence polarization. Horizontal polarization is used in (a) and (b), while vertical polarization is used in (c) and (d). We show the resonance positions via the equation of diffraction conditions, and each line is labeled with the corresponding pair of (i,j). Details can be found in the main text.

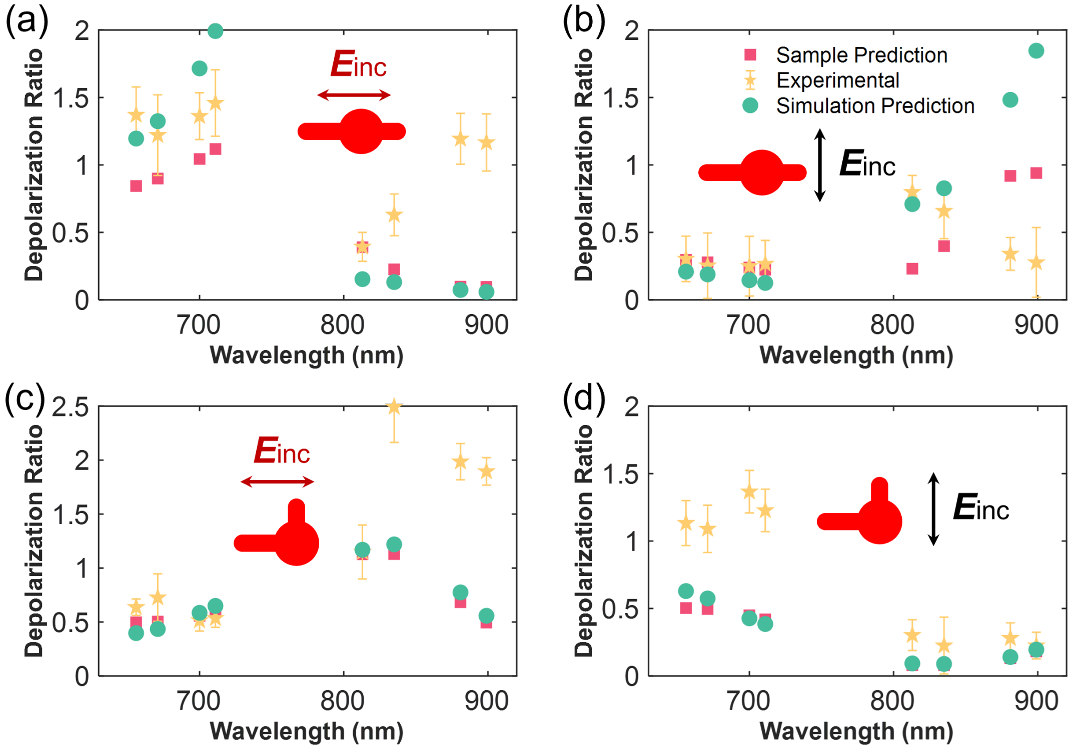

Figure S5: (a-d) SERS depolarization ratio for nanoclock particle arrays with a period of $\Lambda = 500\,\text{nm}$ for four configurations. Orange stars denote the experimental depolarization ratio, while red squares and green dots represent the sample and simulation predictions. The excitation wavelengths are 638 nm (for the left data in the plot) and 785 nm (for the right data in the plot). The inset illustrates the structure and the incident polarization for each case.

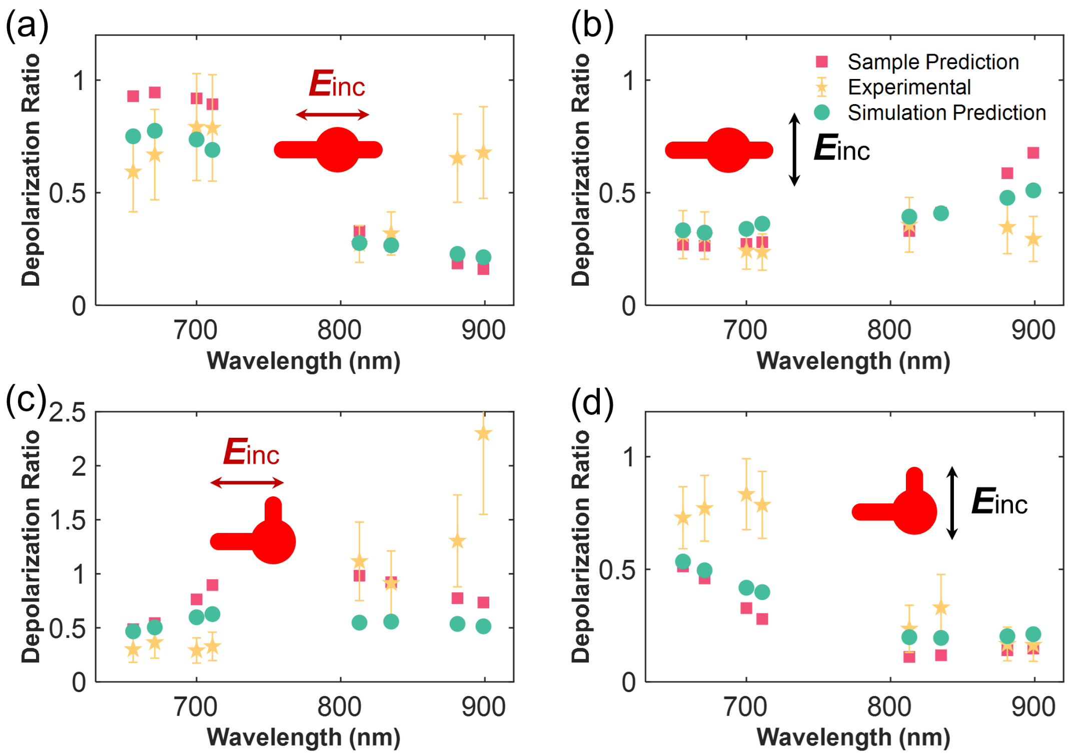

Figure S6: (a-d) SERS depolarization ratio for single structures. The inset shows the structure and the incident polarization for each case. Orange stars indicate the experimental depolarization ratio, while red squares and green dots represent the sample and simulation predictions, respectively. The excitation wavelengths are 638 nm (for the left data in the plot) and 785 nm (for the right data in the plot). The experimental SERS depolarization ratio at the SERS bands agrees reasonably well with the predictions at shorter wavelengths (638 nm excitation), while the agreement worsens at longer wavelengths (785 nm excitation) for both particle arrays and single particles. There could be different possible reasons for this disagreement. First, the extinction signal is much smaller for the longer wavelength, introducing a worse signal-to-noise ratio, which could result in a worse prediction. Second, the figures above are done using the theoretical/experimental extinction, which is the sum of absorption and scattering; the extinction of the gold nanostructure is usually predominated by the absorption at the short wavelengths and the scattering at longer wavelengths. The absorption usually reflects the near-field enhancement, which is more important for the SERS signal. Third, SERS is due to the near-field enhancement of the plasmonic structures, while the extinction spectra are a far-field measurement. It is well-known that there is a shift between the near and far field resonances.[S4,S5] Therefore, using the extinction spectra would introduce uncertainty in predicting the SERS depolarization ratio in some cases.

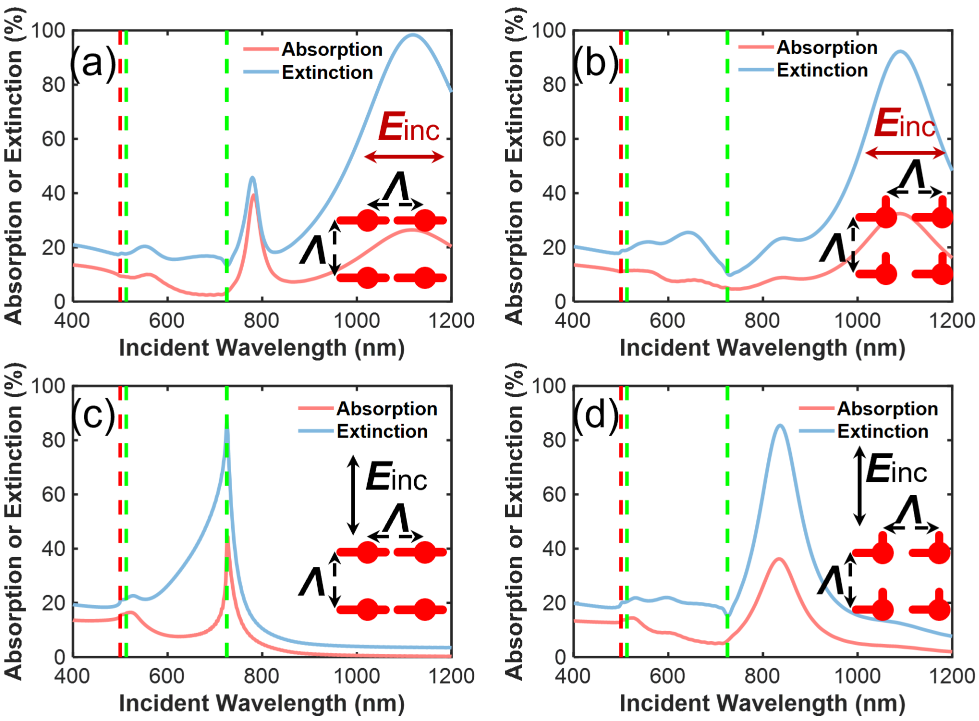

Figure S7: Comparison between theoretical extinction spectra $(1-T)$ and absorption spectra $(1-T-R)$ for Au nanoclock particle arrays. The period $\Lambda = 500\,\text{nm}$ is considered for all four configurations. The schematic is shown in the insets. The shape of the Au nanoclock particles is the same for (a) and (c), while the shape in (b) and (d) is the same. The arrows represent the incidence polarization. Horizontal polarization is used in (a) and (b), while vertical polarization is used in (c) and (d). The resonance positions are shown via the equation of diffraction conditions, with the corresponding (i,j) pairs labeled in Figure S4.

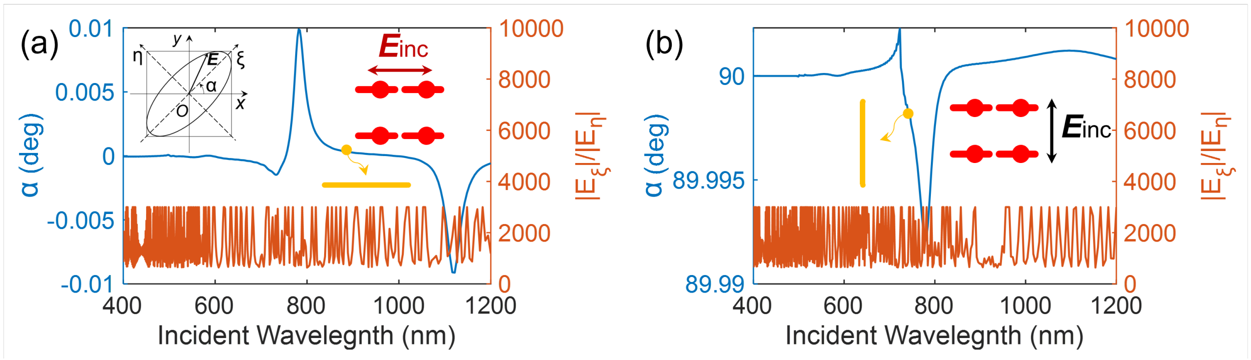

Figure S8: Far-field polarization ellipse calculation for nanoclock particle arrays with $\Lambda = 500\,\text{nm}$ and $\theta = 0°$. The transverse and longitudinal polarization denoted by the arrows is represented in (a) and (b), respectively. In both panels, α and E_ξ/E_η denote the ellipse major axis angle and the major/minor axis ratio, respectively. The noise observed is due to numerical errors. The major/minor axis ratio is above 600 for all wavelengths, indicating that the polarization of the output light remains linear, as expected.

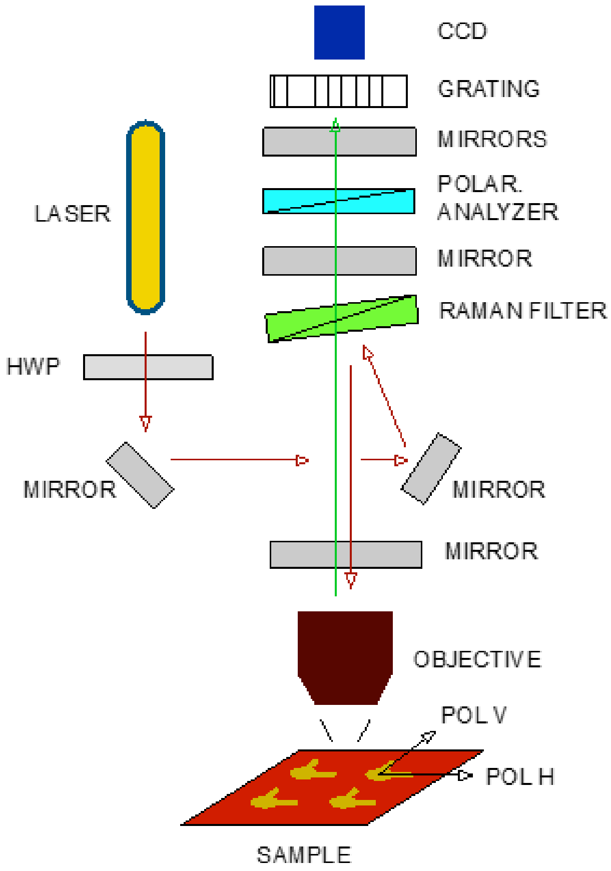

Figure S9: Schematic of the XploRA optical paths for the excitation beam (red arrows) and the SERS/Raman beam (green arrow). Laser light can undergo 90° polarization rotation, thanks to a halfwave plate. Light is thus directed towards the Raman edge filter that reflects it towards the sample. A 100× objective is used to focus the light on the sample surface.

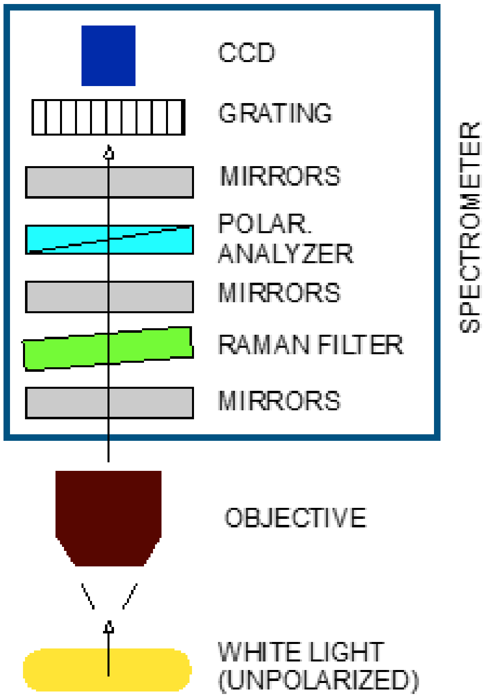

Figure S10: Schematic of the setup used to detect and compensate for the polarization-dependent response of the spectrometer. An LED serves as the light source, and its emission is acquired for two polarization states: horizontal (H) and vertical (V).

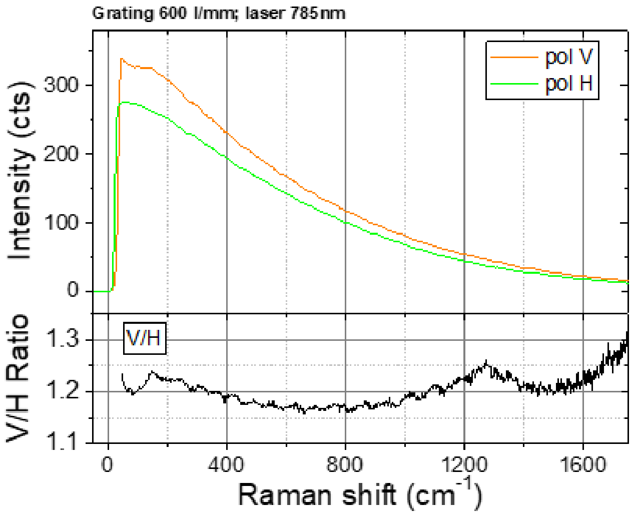

Figure S11: (Top) Spectra of the unpolarized LED light source acquired with the 600 l/mm grating in the range of -100 cm^{-1} to 1750 cm^{-1} relative to the central wavelength of 785 nm, for the H (green line) and V (orange line) polarization states. The flat response at wavenumbers $<$ 50 cm^{-1} is due to the cutting effect of the Raman edge filter. (Bottom) V/H intensity ratio plotted in the same spectral range.

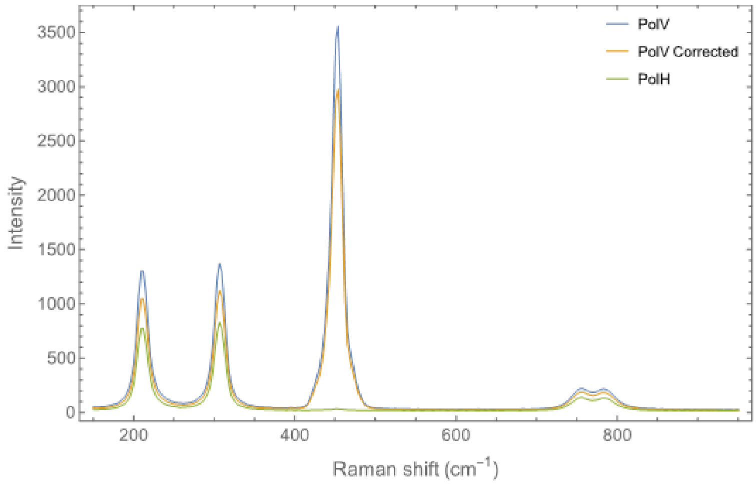

Figure S12: Raman spectra of CCl_4 acquired under two different polarization states, parallel (V, blue line) and cross-polarized (H, green line), using 785 nm excitation. The orange line represents the spectrum after compensation for the instrumental response, referred to as "pol V corrected" spectrum. This compensation step is necessary to obtain the correct depolarization ratio.

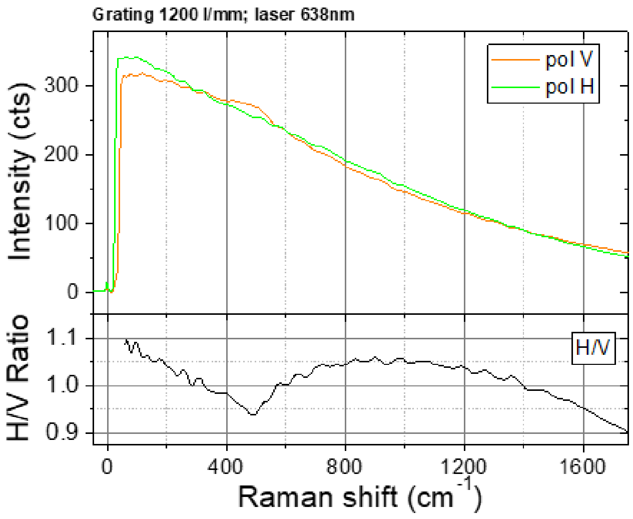

Figure S13: (Top) Spectra of the unpolarized LED light source acquired with the 1200 l/mm grating in the range of -100 cm^{-1} to 1750 cm^{-1} relative to the central wavelength of 638 nm, for the H (green line) and V (orange line) polarization states. The flat response at wavenumbers $<$ 50 cm^{-1} is due to the cutting effect of the Raman edge filter. (Bottom) H/V intensity ratio plotted in the same spectral range.

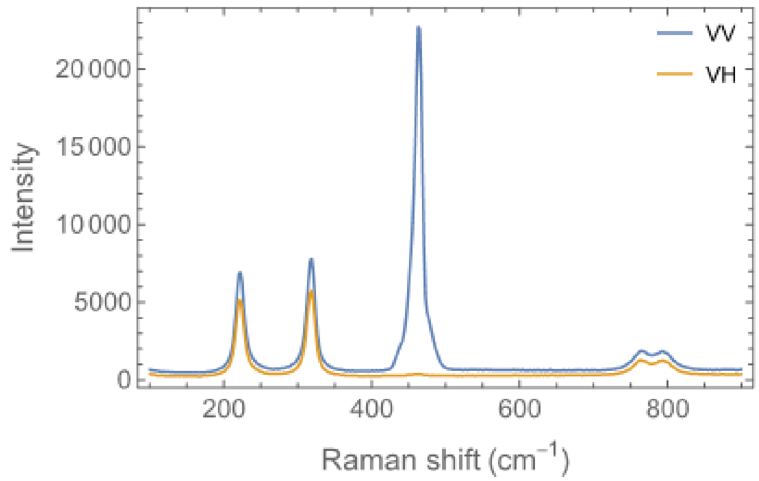

Figure S14: Raman spectra of CCl_4 acquired at 638 nm excitation in parallel- (VV, blue line) and cross-polarized states (VH, orange line). Spectra are offset for clarity.

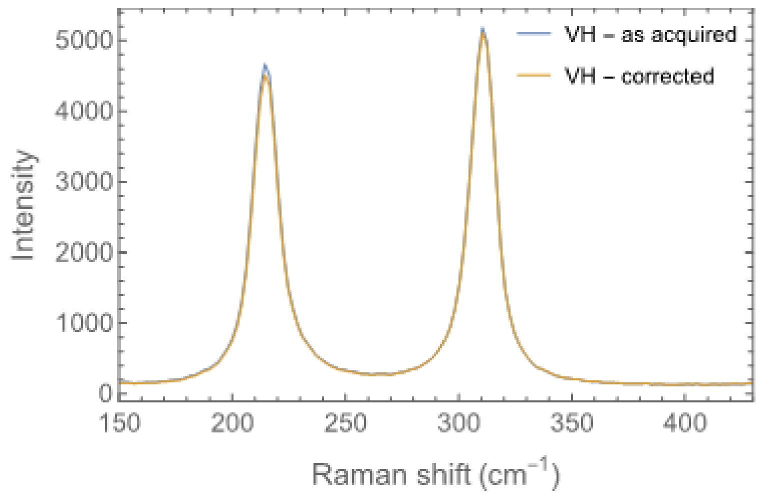

Figure S15: As acquired (blue line) and corrected (orange line) Raman spectrum of CCl_4 acquired at 638 nm excitation in the cross-polarized state.

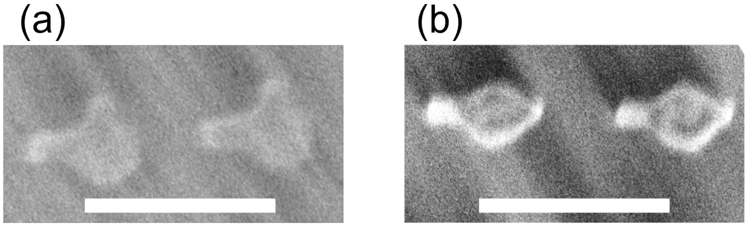

Figure S16: Scanning electron microscopy images of Au nanoclock arrays, when $\Lambda = 500\,\text{nm}$. (a) $\theta = 90°$ and (b) $\theta = 180°$. The scale bars represent 500 nm

References

(S1) Adar, F. Raman Polarization Measurements: Keeping Track of the Instrumental Components' Behavior. `https://www.spectroscopyonline.com/view/raman-polarization-measurements-keeping-track-instrumental-components-behavior`, 2017; (accessed on 1/March/2023).

(S2) Fazio, B.; D'Andrea, C.; Bonaccorso, F.; Irrera, A.; Calogero, G.; Vasi, C.; Gucciardi, P. G.; Allegrini, M.; Toma, A.; Chiappe, D., *et al.* Re-radiation enhancement in polarized surface-enhanced resonant Raman scattering of randomly oriented molecules on self-organized gold nanowires. *Acs Nano* **2011**, *5*, 5945–5956.

(S3) Johnson, P. B.; Christy, R.-W. Optical constants of the noble metals. *Physical review B* **1972**, *6*, 4370.

(S4) Colas, F. J.; Cottat, M.; Gillibert, R.; Guillot, N.; Djaker, N.; Lidgi-Guigui, N.; Toury, T.; Barchiesi, D.; Toma, A.; Di Fabrizio, E., *et al.* Red-shift effects in surface enhanced Raman spectroscopy: spectral or intensity dependence of the near-field? *The Journal of Physical Chemistry C* **2016**, *120*, 13675–13683.

(S5) Zuloaga, J.; Nordlander, P. On the energy shift between near-field and far-field peak intensities in localized plasmon systems. *Nano letters* **2011**, *11*, 1280–1283.
